# Electrical excitation of self-hybridized exciton polaritons in a van der Waals antiferromagnet

**DOI:** 10.1126/sciadv.adz6724

**Published:** 2025-11-07

**Authors:** Jonas D. Ziegler, Sotirios Papadopoulos, Antti J. Moilanen, Marcelo Martínez, Qia Lin, Kseniia Mosina, Takashi Taniguchi, Kenji Watanabe, Zdenek Sofer, Florian Dirnberger, Lukas Novotny

**Affiliations:** ^1^ Photonics Laboratory, ETH Zürich, Zürich 8093, Switzerland.; ^2^ Institut de Physique et Chimie des Matériaux de Strasbourg, Université de Strasbourg, CNRS, Strasbourg UMR 7504, France.; ^3^ Department of Inorganic Chemistry, University of Chemistry and Technology Prague, Prague 166 28, Czech Republic.; ^4^ International Center for Materials Nanoarchitectonics, National Institute for Materials Science, Tsukuba, Ibaraki 305-004, Japan.; ^5^ Research Center for Functional Materials, National Institute for Materials Science, Tsukuba, Ibaraki 305-004, Japan.; ^6^ Zentrum für Quantum Engineering (ZQE), Technical University of Munich, Garching, Germany.; ^7^ Physics Department, TUM School of Natural Sciences, Technical University of Munich, Munich, Germany.; ^8^ Munich Center for Quantum Science and Technology (MCQST), Technical University of Munich, Garching, Germany.

## Abstract

The coupling of light with excitations in matter is one of the most important concepts to make photons interact, crucial for the development of efficient optoelectronic devices. In materials with exceptionally strong light-matter interaction, excitons can hybridize with photons without the need of an external cavity. Here, we report the electrical excitation of such self-hybridized polaritons in the van der Waals antiferromagnet CrSBr. We exploit an unconventional excitation via energy transfer from tunneling electrons in graphene tunnel junctions to strongly bound excitons in proximate CrSBr layers. This enables us to excite CrSBr crystals ranging in thickness from a bilayer up to 250 nanometers, with the strong linear polarization of the electroluminescence confirming the excitonic origin. We assign the electrically excited emission to self-hybridized exciton polaritons, highlighting the strong coupling between optical excitations and confined photon modes in CrSBr. Our findings not only offer an efficient method to generate polaritons electrically but also create opportunities for future spintronic devices.

## Teaser

A near-field energy transfer excites hybrid light matter particles in the layered magnet CrSBr up to room temperature.

## INTRODUCTION

Two-dimensional (2D) materials have emerged at the forefront of condensed matter research, providing a versatile platform to explore novel quantum phenomena and enabling potential applications in next-generation devices. Within this realm, van der Waals (vdW) magnets are particularly attractive, as their weak interlayer interactions facilitate precise atomic-scale confinement down to the monolayer limit (*1*–*3*). In addition to metallic and semimetallic vdW materials, semiconducting vdW magnets have attracted considerable attention owing to their tunable optical and electronic properties, making them promising candidates for fundamental research (*4*–*7*) and spintronic applications (*8*, *9*).

Among these materials, chromium sulfur bromide (CrSBr) has rapidly gained prominence due to its compelling combination of desirable properties (*10*–*14*). CrSBr exhibits a relatively high Néel temperature (*15*), air stability, and strongly bound excitons with large oscillator strengths (*16*). Furthermore, its intrinsic antiferromagnetic (AFM) ordering and strong electronic anisotropy render it particularly suitable for applications in quantum technologies and spintronics (*17*, *18*).

A particularly intriguing characteristic of CrSBr lies in the interplay between its optical excitations and magnetic ordering. The strong oscillator strength in CrSBr allows for the formation of self-hybridized exciton polaritons, enabling unprecedented control and optical tunability of excitonic states (*19*). These unique light-matter interactions provide new opportunities to manipulate quantum states through optical methods.

However, effectively exploiting these optical and spintronic features for practical device applications requires electrical excitation and control of excitonic states. Traditionally, electrical excitation in conventional 2D semiconductors is achieved through separate injection of electrons and holes (*20*), a mechanism critical for realizing efficient spin-based devices and coherent quantum control.

In this work, we introduce an electrical excitation mechanism in CrSBr, mediated by the coupling of tunneling electrons to excitons, that occurs independently of flake thickness. Specifically, we demonstrate electrically excited excitonic states in CrSBr flakes ranging from bilayer (1.6 nm) thickness up to bulk crystals (250 nm). Notably, thicker samples exhibit multiple resonances consistent with previously observed self-hybridized exciton polaritons (*19*, *21*). This near-field excitation mechanism, along with the unconventional magnetic response of the material, opens exciting pathways for the manipulation of excitonic and electronic states in magnetic vdW materials.

## RESULTS

### Energy transfer excitation from tunneling electrons

**Fig. 1.**
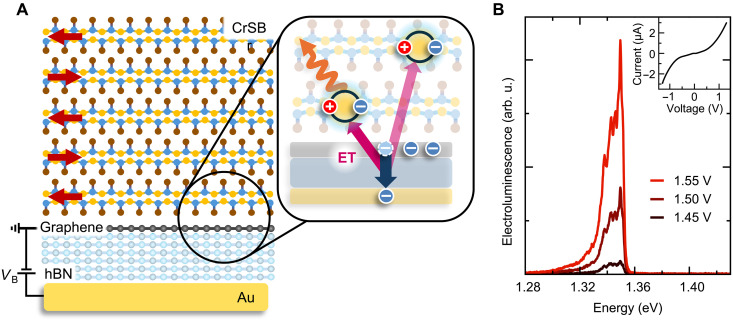
Energy transfer excitation in CrSBr. (**A**) Schematic illustration of the device featuring a CrSBr layer on top of a graphene–hexagonal boron nitride (hBN)–gold tunneling junction. Red arrows indicate the AFM order of CrSBr below the Néel temperature. For electroluminescence (EL), the graphene layer is grounded, and a bias voltage *V*_B_ is applied to the gold layer across the hBN. Zoom-in view depicts the exciton generation via an energy transfer (ET) from a tunneling electron to excitons in the proximate lower layers of the CrSBr. (**B**) EL from a 25-nm-thick CrSBr flake for increasing voltage. Inset shows the corresponding tunneling current as function of the applied voltage. arb. u., arbitrary units.

We use a recently reported near-field excitation technique driven by tunneling electrons for CrSBr (*22*, *23*). In this design, the active material is positioned outside the direct electrical conduction pathway. This forms an open electrode configuration, similar to open cavity designs. Figure 1A illustrates the device design, which consists of a CrSBr layer of variable thickness placed on top of a graphene–hexagonal boron nitride (hBN)–gold tunnel junction.

The mechanism underlying the electrical excitation, based on near-field optical processes, is illustrated in the inset of Fig. 1A. When a voltage is applied between the gold and graphene electrodes, electrons tunnel from graphene into the gold electrode. This tunneling requires conservation of both energy and momentum (*24*). Electrons undergoing elastic tunneling enter high-energy states in gold and, subsequently, dissipate energy through interactions with phonons within the metal. Conversely, electrons undergoing inelastic tunneling can directly emit photons, generating broadband radiation corresponding to their energy loss (*25*–*27*). When a strong dipolar excitation is nearby, tunneling electrons can alternatively transfer their energy directly to this excitation (*28*). The efficiency mainly depends on three factors: distance, oscillator strength of the exciton, and its dipole orientation. Here, the exceptionally strong exciton oscillator strength of CrSBr (*19*) and the vdW nature make it a highly promising material for this excitation mechanism.

In this scenario, tunneling electrons couple to excitons in adjacent layers, thereby directly exciting excitons in the neighboring material. Our measurements do not provide evidence for direct charge-carrier injection into the CrSBr layer, as further supported by the comparison with a field-effect device presented in the Supplementary Materials (see fig. S5). In particular, direct tunneling into the conduction or valence band states of CrSBr would be expected to produce an additional increase in current and, therefore, a conductance peak, neither of which are observed in our data. This method offers distinct advantages: Excitons can be excited without applying an electric field directly across the active material, enabling the study of intrinsic excitonic properties and avoiding degradation in sensitive samples. Moreover, direct exciton generation prevents charge carrier imbalance. Last, this “open electrode” design excites excitons, irrespective of the CrSBr thickness, as the CrSBr layer is placed on top of the tunnel junction.

The devices are fabricated using a dry-pick up method (*29*), stacking mechanically exfoliated CrSBr, graphene, and thin (four to seven layers) tunneling hBN. All fabrication steps for handling CrSBr are performed inside an argon-filled glove box to prevent oxidation and ensure clean interfaces. Devices are further capped with an hBN layer to protect the devices from the environment for the measurement (*30*, *31*). The final stack is released on prepatterned gold contacts, which serve both as the bottom tunneling layer and the contact for the graphene layer (see Materials and Methods and fig. S1 for more details). For electroluminescence (EL) measurements, we apply a voltage between the graphene and the bottom gold layer across the thin tunneling hBN. An exemplary EL spectrum acquired at cryogenic temperatures is shown in Fig. 1B for a 25-nm-thick CrSBr flake on top of a tunneling junction. The emission spectrum exhibits a pronounced resonance near 1.35 eV, with related smaller features appearing at lower energies. The EL intensity strongly increases with increasing voltage. Similarly, the corresponding IV curve (see inset of Fig. 1B) exhibits a clear tunneling behavior, with an exponentially increasing current at high voltages.

### Electrically excited excitons in CrSBr

**Fig. 2.**
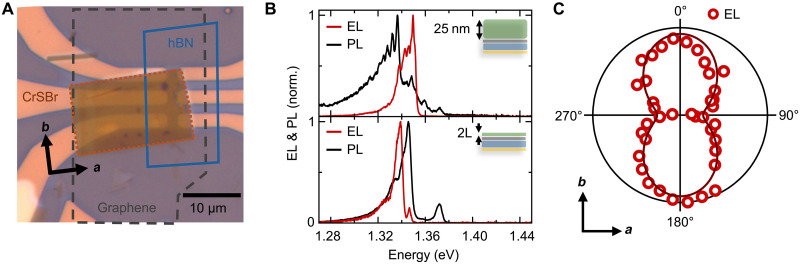
Electrically excited excitons in CrSBr. (**A**) Optical microscope image of a tunneling junction with 25-nm-thick CrSBr layer on top. Top left shows additional flake transferred during stacking, with the typical needle-like shape indicating the crystallographic *a* and *b* axes. (**B**) Comparison of EL and PL for a thick flake (25 nm, top) and a bilayer (2L; bottom) CrSBr. (**C**) Linear polarization of the EL as polar plot, with *a* and *b* axes indicated. Dark red line is a cos^2^ fit to the data.

An exemplary device is shown in the optical microscope image in Fig. 2A. The open electrode design enables the use of arbitrary thickness of the CrSBr; the one in the image is around 25-nm (around 30 layers) thick. It covers part of the graphene-hBN-gold tunnel junction on the right side. The EL of this device is shown in the top part of Fig. 2B together with the respective photoluminescence (PL) spectrum (black). The electrically excited emission (red) is dominated by a main resonance at an energy of 1.35 eV with additional peaks at lower energy. The periodicity of the additional features is similar to the shape of the PL and might indicate an origin related to phonon replicas. Different spacings have been reported in literature, whereas the observed spacing of around 4 meV is on the lower order (*13*).

The main peak of the EL coincides with a smaller feature in the PL, while the high energy features are missing in the EL. The change in intensity distribution can be attributed to the specific excitation mechanism: In PL, excitons are homogeneously generated throughout the sample by a red laser with an excitation energy exceeding the emission energy by more than 0.5 eV. Consequently, excitons must relax to lower-lying states prior to recombination; however, radiative recombination can occur during this relaxation process, depending on the exciton lifetime and the occupancy of intermediate states. In EL on the other side, only layers in close proximity to the tunneling junction are excited by the near-field excitation. Excitons are generated resonantly by the energy transfer, making the EL additionally more sensitive to the absorption of the distinct states. This changes the overall coupling to different states and modes in the system.

However, instead of a change in intensity distribution, the optically and electrically excited emission can originate from different states. The lowest-lying layers experience a different dielectric environment because of the graphene layer, leading to screened Coulomb interactions and a change in exciton energy (*14*). In addition to the dielectric surrounding, the magnetic domains also change markedly at the interface. Recently, surface excitons have been reported in CrSBr few layers, which exhibit magnetic confinement to the surface layer (*32*). Due to the strong distance dependence of the near-field excitation, surface and bulk states could potentially influence the distribution. To gain more insight into the contribution from bulk and surface excitons, we further investigate this in a bilayer device, shown in the bottom panel of Fig. 2B. Here, the PL (black) exhibits both bulk and surface exciton at energies of 1.37 and 1.34 eV, respectively. Consequently, the EL of the bilayer is more similar to the PL and mainly consists of one resonance slightly below 1.34 eV, which we attribute to the strong surface exciton. The remaining slight blueshift of the EL compared to that of the PL can be a redistribution of the two visible features above and below 1.34 eV. The homogeneous hyperspectral PL maps shown in figs. S6 and S7 rule out spatial inhomogeneities as the origin of the observed differences between EL and PL. We observe the bulk exciton state in the PL response of the bilayer. This counterintuitive observation is in agreement with recent literature and can be understood by looking at the crystal structure of a single CrSBr layer: a bilayer of chromium sulfide layers encapsulated by the bromide atoms. This leads to the formation of both a bulk like state in the center and two surface states on the outside for the bilayer. The bulk state is missing in the EL, a direct consequence of the near-field excitation and the additional distance of the bulk state from the excitation dipole.

Moreover, we observe a strong polarization anisotropy of the EL, as shown in the polarization-resolved measurement in Fig. 2B. This is a clear hallmark of excitons in CrSBr, where the quasi-1D band structure leads to a strong anisotropy of the optical resonance (*10*, *15*). Consequently, the high-intensity axis aligns well with the *b* axis of the crystal. This gives further evidence of the electrical excitation of excitons in CrSBr flakes ranging from bilayer to bulk. It is relevant to point out that we do not observe evidence for charged exciton states, independent of sample thickness and bias polarity (*33*). The trion states are expected to appear around 20 meV below the main exciton resonance, while the main peak of the EL is more than 30 meV below the highest PL state. We show further evidence in fig. S5 by comparing the increase in tunneling bias with a gate tunable bilayer, which shows the charged excitons in positive and negative polarity. This shows that the near-field energy transfer directly excites charge-neutral excitons, contrasting conventional charge carrier injection mechanisms.

### Self-hybridized exciton polaritons in bulk CrSBr

**Fig. 3.**
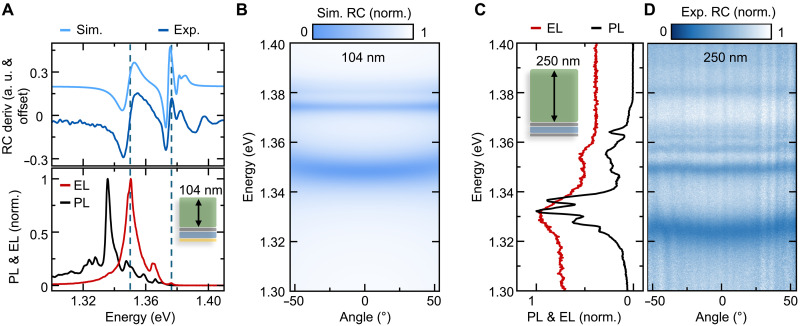
Self-hybridized exciton polaritons in thick CrSBr. (**A**) Comparison of the reflectance (top) with the emission (bottom) of a 104-nm-thick flake. Reflectance contrast derivative (RC deriv) is depicted in dark blue, corresponding simulated response in light blue. Main resonances of EL (red) and PL (dark blue) are well matched with the absorption-type measurement. a. u., arbitrary units. (**B**) Angle-resolved simulation of the reflectance for a sample matching the experimental device, where we assume a single exciton resonance at 1.387 eV. The additional resonances are due to the coupling between the light modes inside the CrSBr flake and the strong exciton resonance. Simulation in (A) is the derivative of the angle-integrated spectrum. (**C**) EL and PL spectra for a 250-nm-thick CrSBr flake on top of graphene-hBN-graphene tunnel junction. (**D**) Experimental angle-resolved reflectance contrast of the 250-nm-thick CrSBr, exhibiting clear bending of the low-energy modes.

We now turn to the emission of a 104-nm-thick layer to gain further insight into the coupling to optical modes of the CrSBr slab. In Fig. 3A, we show the EL together with the PL and the derivative of the reflectance contrast RC deriv (blue). EL and PL are slightly shifted while exhibiting the same shape, which consists of multiple peaks. This is distinctly different from the bilayer case and already observable in the PL of the 25-nm-thick sample. The main peaks of the EL correspond to resonances observed in the reflectance contrast, indicated by the dashed lines in Fig. 3A. This is a clear signature for self-hybridized polariton states recently reported, where each peak is one polariton branch (*19*, *21*). Here, the bare crystals confine photons due to the marked change in refractive index at the interface with air on one side and the gold electrode on the other side.

The individual modes are well captured by the simulated reflectance in Fig. 3B, which is shown as function of incident angle of the light. We model the optical response of the sample by taking all the layers into account in a transfer matrix model, where we estimate the dielectric function of CrSBr with a single exciton resonance. The angle-dependent bending of the resonances highlights the polaritonic nature, whereas the overall small effect emphasizes the strong excitonic part of the observed polaritons for this thickness. The evolution of the angle-resolved spectrum with increasing CrSBr thickness shown in fig. S3 further illustrates the importance of the photonic environment. Figure 3C presents the emission spectra of a bulk CrSBr flake (250 nm thick, see fig. S4 for more details) on top of a graphene-hBN-graphene electrode structure. The overall spectral shape of the EL closely resembles that of the corresponding PL, although the individual resonances appear less sharp in comparison. This is consistent with the different excitation mechanisms involved, especially regarding the EL stemming from the lowest few layers inside the flake. The reflectance contrast spectrum of the same device, shown in Fig. 3D, reveals a clear bending of the optical modes, especially for the lowest energy mode. These features directly correspond to the resonances observed in both EL and PL, thereby confirming the consistency between absorption- and emission-based measurements and providing further evidence for the hybrid excitonic nature of the observed states.

### Coupling of inelastic electron tunneling

**Fig. 4.**
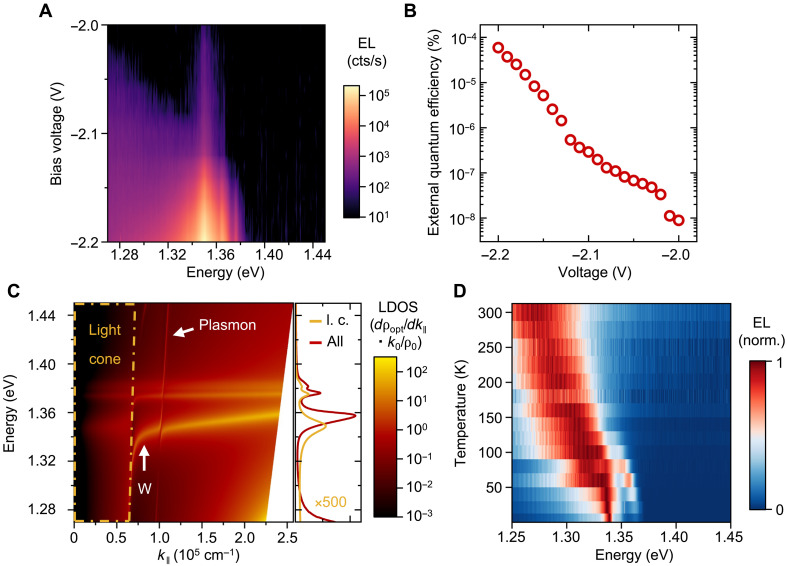
Voltage and temperature dependence of EL. (**A**) Voltage dependent EL of a 104-nm-thick CrSBr on top of a graphene-hBN-gold junction. cts, counts. (**B**) External quantum efficiency (EQE; electron-to-photon conversion efficiency) as function of the applied voltage. (**C**) Angular spectral density of the LDOS for an out-of-plane dipole placed 2 nm away from a 104-nm-thick CrSBr flake (as schematically depicted in inset of Fig. 1A). The dashed-dotted line with anticrossing features close to *k*_ǁ_ = 0.75 × 10^5^ cm^−1^ resembles the light cone (l. c.) of the material. Additionally, the feature at around *k*_ǁ_ = 1 × 10^5^ cm^−1^ is assigned to the plasmon resonance between graphene and the gold bottom electrode. In between these features, a highly dispersive mode is characterized as the waveguide mode (W). Right side shows angle-integrated spectra for the light cone (yellow, ×500) and the full angular spectrum (red). (**D**) Temperature dependence of the EL signal from 5 to 300 K, normalized for each temperature.

Having established the electrical excitation of exciton polaritons via a near-field energy transfer, we will now discuss the efficiency of this process. The electrically excited emission as a function of voltage is shown in Fig. 4A. Here, multiple resonances are observed around 1.36 eV, while the overall emission intensity increases strongly. We also observe weak, broadband emission at lower energies, which we attribute to the direct coupling of tunneling electrons to photons. The coupling to excitons is expected to be much more efficient than the coupling to photons due to the relaxed momentum conservation. Here, excitons can be excited with high momentum compared to photons limited to the light cone. This is directly observable in the strong exciton emission of Fig. 4A.

The external quantum efficiency (EQE), shown in Fig. 4B, is overall low but exhibits a marked increase as the applied voltage rises. Here, the presented EQE is a lower bound on the efficiency, as the CrSBr covers only partially the tunneling area. This trend is primarily attributed to the relative enhancement of inelastic tunneling versus elastic tunneling at higher voltages, which facilitates greater electron-exciton coupling (*34*). For the investigated device, we also observed EL only at elevated voltages, but, in principle, we would expect to see emission even below the bandgap (*35*). Nevertheless, the observed efficiency remains limited due to notable quenching induced by graphene, which likely varies with the Fermi level of graphene (*36*, *37*). The complex interface between CrSBr and graphene also introduces variability in the charge and energy transfer dynamics (*38*). Furthermore, the near-field excitation creates a highly inhomogeneous exciton population with most of the excitation happening in a few layers in in close proximity of the graphene. This strong density gradient will distribute throughout the stack by vertical exciton diffusion, affecting the overall efficiency. Comprehensive investigations into exciton transport mechanisms in CrSBr will be required to clarify these processes.

Exciton generation is directly tied to the inelastic tunneling rate (*22*, *24*), which depends on the local density of optical states (LDOS), plotted in Fig. 4C. In our samples, the LDOS is significantly enhanced due to the high oscillator strength and the formation of self-hybridized exciton polaritons. This enhancement enables tunneling electrons to couple efficiently with excitonic states, notably even at elevated electron momenta. The LDOS is calculated by placing a dipole in the center of our tunneling diode and determining the radiated power by taking the real sample geometry of the 104-nm-thick flake into account (see the Supplementary Materials and fig. S2 for the detailed sample structure). The rich LDOS evidences the formation of self-hybridized polaritons inside the light cone (yellow dashed area) and beyond inside the material. It further shows the existence of a strong waveguide mode (W) between the two CrSBr facets, which can be connected to the presence of hyperbolic exciton polaritons (*16*). If we consider the LDOS inside the light cone separately by integrating the angular contributions as displayed in the right side of Fig. 4C, then a clear separation in energy can be observed. This is reminiscent of the measured PL and EL spectra, where the difference can alternatively be explained by the electrical excitation of high-angle modes, which cannot be addressed optically. The modes with strong in-plane character then contribute to the observed emission via scattering at the gold edges, thus changing the overall emission characteristics when exciting electrically.

It is crucial to note that, although we expect plasmon excitation originating from the gold, identified by the pronounced, nearly vertical dispersion line around *k*_ǁ_ = 1 × 10^5^ cm^−1^ in Fig. 4C, this plasmonic excitation is not fundamentally required for the observed EL. Similar EL behavior from exciton polariton states is observed in graphene-hBN-graphene tunneling devices entirely without gold in the emission area (see Fig. 3, C and D).

Exploring the temperature dependence, Fig. 4D distinctly reveals electrical excitation above the Néel temperature of ~130 K and up to room temperature. The transition is accompanied by a simultaneous decrease in oscillator strength and an increased linewidth at the critical temperature (*32*). Above this transition temperature, direct exciton emission notably reduces, and weak broadband emission emerges from direct electron-light coupling processes at higher energies (see fig. S8 for additional temperature dependence on the 25-nm device). Observing emission at room temperature is a first step toward the practical relevance and applicability of these materials for future optoelectronic devices. This capability could greatly facilitate their integration into realistic device architectures beyond CrSBr, paving the way for versatile technological applications.

## DISCUSSION

In summary, we show electrical excitation of exciton polaritons in a vdW magnet with variable layer thickness from cryogenic up to room temperature. For thicker crystals, self-hybridized exciton polariton states are the main excitation modes, highlighting the strong light-matter interaction in CrSBr. We establish energy transfer from tunneling electrons to excitons as an alternative to conventional charge injection, thereby introducing a previously unidentified strategy for device design. This excitation mechanism also enables the in situ study of interfaces with other materials (*38*–*41*), without the need of external optical excitation. In particular, the expected excitation of a variety of modes ranging from high-angle modes inside the material, hyperbolic exciton polaritons in CrSBr, and the surface plasmon polariton at the interface with graphene offer promising research directions.

The overall low efficiency of the device presents an opportunity for further improvement, where one reason is the strong excitation of dark modes beyond the light cone, as seen in Fig. 4C. Here, the enhancement of the outcoupling efficiency of these modes by additional structures such as gratings poses an interesting direction, which can further provide insights into the electrical excitation. For multiple investigated CrSbr thicknesses, we observe a distinct difference in EL and PL spectra, which are also identified in similar studies (*42*, *43*). We attribute this change to both the different optical environment and the role of surface and bulk excitons, whereas deeper understanding of the underlying excitation differences is needed. From an electrical point of view, the elastic tunneling rate needs to be reduced by engineering the electrode materials to make more electrons couple to exciton polaritons. Last, the strong coupling between excitons and the magnetic order makes electrical excitation an important tool for future applications. Our work opens new avenues toward direct readout of magnetic states, paving the way for simpler device architectures.

## MATERIALS AND METHODS

### Device fabrication

High-quality hBN flakes (National Institute for Materials Science, Japan) and commercially available graphite (NGS trading) are exfoliated on Si substrates with a 100-nm SiO_2_ layer, which has been treated with oxygen plasma. The thickness of the tunneling hBN is verified with an atomic force microscope. CrSBr (Prague) is exfoliated on Si substrates with a 100-nm SiO_2_ layer without plasma treatment, as especially thin CrSBr are challenging to be transferred. The flake thickness of CrSBr is identified first by contrast and verified with an AFM measurement of the finished device. A thin layer of polycarbonate (PC) is placed on a polydimethylsiloxane (PDMS) stamp for the dry pickup. First, a thick (15 to 40 nm) hBN layer is picked up as top layer, which is used to subsequently pick up the CrSBr, graphene, and tunneling hbN. Last, the PC layer with the stack is released onto prepatterned gold electrodes (50-nm gold with a 5-nm chromium layer for better adhesion). Last, the PC film is dissolved in chloroform and isopropanol. For the 250-nm-thick flake, a tunnel junction made only from graphene, tunneling hBN, and another graphene layer is stacked first as described earlier and tested. Then, the thick CrSBr is exfoliated on a PDMS layer and transferred on top of the tunnel junction.

### Electrical and optical measurements

All electrical and optical measurements are performed inside a closed-cycle cryostat (Attodry 800). Electrical measurements are performed using an amperemeter (Keithley 6482), which is also used as bias source. For PL measurements, the sample is excited with a continuous wave helium-neon laser with a photon energy of 1.96 eV. Emitted light is detected either directly by an electron multiplying charge-coupled device (Andor iXon 897) or spectrally dispersed by a spectrometer (Andor SR303) and detected by a charge-coupled device camera (Andor iDus 416). For linearly polarized measurements, the sample is driven by same bias voltage, while a linear polarizer placed in the detection path is rotated. The EQE of the sample is defined as the number of photons emitted per number of injected electrons, for which transmission spectrum measurements are performed to calibrate the absolute collection efficiency of the optical setup. A halogen calibration light source with calibrated spectrum (OceanOptics HL-2000) is used to obtain the shape of the transmission spectrum. Then, the same excitation laser is used to get the absolute value of the transmission at photon energy of 1.96 eV, which corrects the transmission spectrum obtained by the halogen light source to an absolute position. From this, we can estimate the absolute number of emitted photons during the exposure time, which is divided by the total measured current through the junction. Considering the finite slit-width of the spectrometer and the nonuniform angular photon emission of the sample and the objective with numerical aperture of 0.81, our estimation yields the lower bound of the EQE.

### Reflectance and LDOS calculations

The LDOS $\rho_{\text{opt}}$ , relative to the vacuum density of states $\rho_{0}$ , is calculated from the radiated power of a dipole P , according to (*44*): $\frac{\rho_{\text{opt}}}{\rho_{0}}=\frac{P}{P_{0}}$ , with $P_{0}$ being the power dissipated for a point dipole in vacuum. The dissipated power *P* is calculated from $P=\frac{1}{2}\omega \;p\;\text{Im}\left\{\;E\left(r_{0}\right)\;\right\}$ , with the emitted dipole angular frequency ω and the dipole moment p . The dipole origin $r_{0}$ is placed at the center of the tunneling hBN, and wave equations are solved to calculate the electric field E . The response from the sample was then obtained from the standard Fresnel formalism, see fig. S2 for more details. The refractive indices were obtained from (*19*).

## Supplementary Materials

### This PDF file includes:

Supplementary Text Figs. S1 to S8 References
