# Supplementary material for: Electrical excitation of self-hybridized exciton polaritons in a van der Waals antiferromagnet
Source: Sci Adv. 2025 Nov 7;11(45):eadz6724. doi: 10.1126/sciadv.adz6724 (PMC12594166; doi:10.1126/sciadv.adz6724)
Supplement: Supplementary file 1 — Supplementary Text Figs. S1 to S8 References [file sciadv.adz6724_sm.pdf]

Science Advances
AAAS

Supplementary Materials for

Electrical excitation of self-hybridized exciton polaritons in a van der Waals antiferromagnet

Jonas D. Ziegler *et al.*

Corresponding author: Jonas D. Ziegler, zieglerj@ethz.ch

Sci. Adv. **11**, eadz6724 (2025)
DOI: 10.1126/sciadv.adz6724

This PDF file includes:

Supplementary Text
Figs. S1 to S8
References

Supplementary Information and data

Sample fabrication

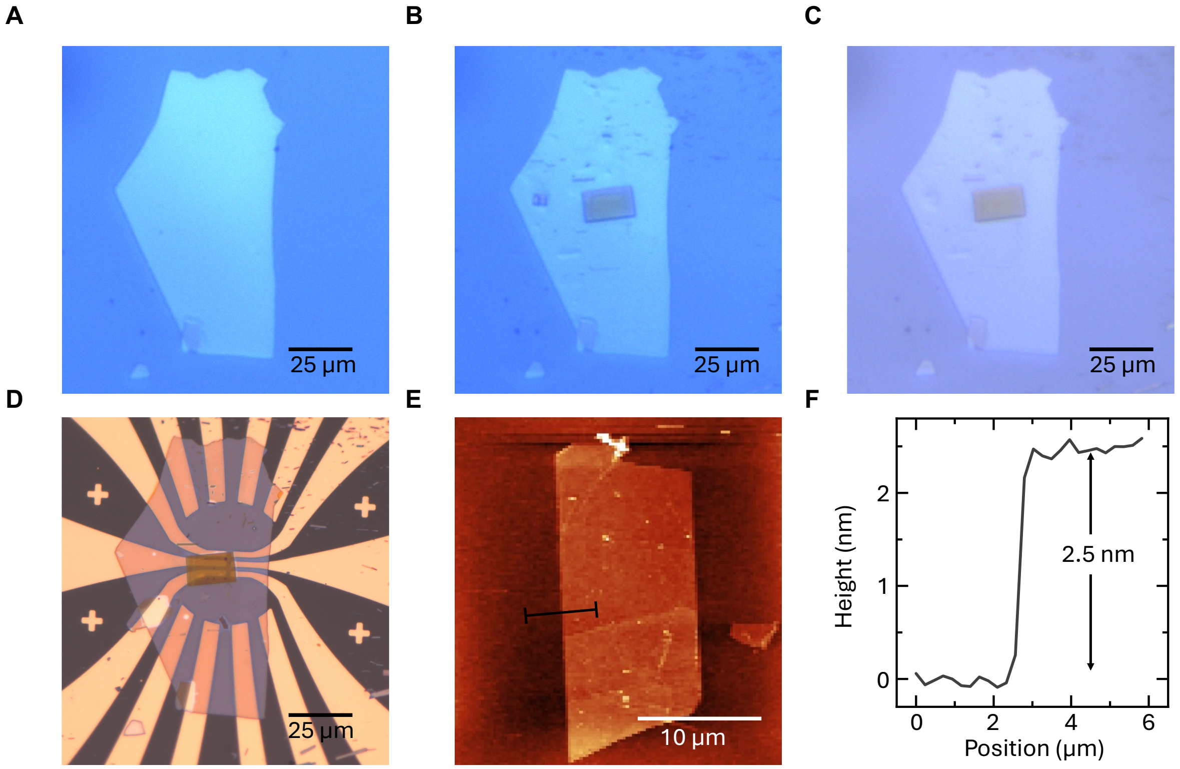

Figure S1: **Step-by-step stacking.** **(a)** Top hBN layer picked up by a polycarbonate (PC) film on a polydimethylsiloxane (PDMS) stamp. **(b)** Picked up CrSBr layers, the rectangular layer in the center the device layer. Additional CrSBr flakes exhibit the typical needle-like shape indicating the crystallographic a- and b-axis. **(c)** Stamp after adding the graphene layer to the stack. Due to the limited contrast, the graphene layer is barely visible. **(d)** Finished device dropped down on pre-patterned gold electrodes (50 nm gold above 5 nm Chrome). The tunneling hBN covers the right side of the graphene, making the overlap region on the right side the active device area. **(e)** Atomic force microscope image of the employed tunneling hBN. **(f)** Height profile along the black line in (e), averaged over multiple pixel.

Calculations of optical density of states

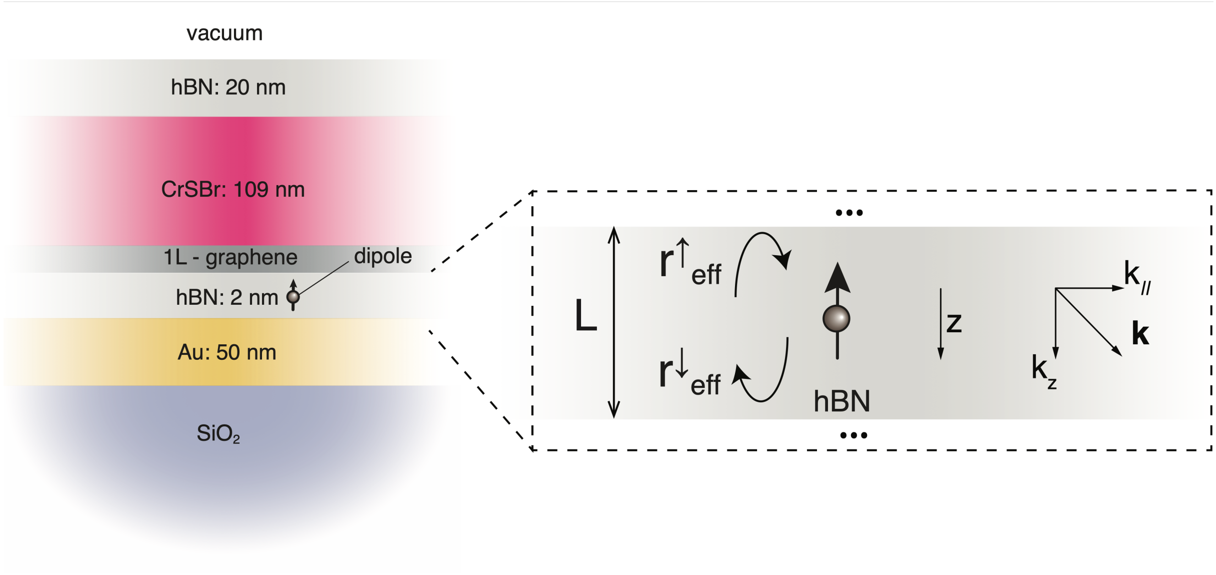

Figure S2: **Sample structure employed for LDOS calculations.** Schematic of the sample shown in main figure 3 and used for LDOS calculations. The dipole is situated in the center of the tunneling hBN, estimated with a thickness of 2nm. A thickness of 109 nm for the CrSBr yields the best fit to the data, in good agreement with the experimental thickness of 104 nm, measured using an atomic force microscope.

We calculate the LDOS for a vertical dipole at the mid-plane of the tunneling hBN layer in the stack shown in fig. S2. The LDOS is obtained from the total dissipated (*44*)

$$\mathcal{P} = \frac{\omega}{2}\,\mathrm{Im}[\mathbf{p}^{*}\cdot\mathbf{E}_{\mathrm{tot}}(\mathbf{r}_{d})]\,, \tag{S1}$$

where $\mathbf{p}$ is the dipole moment, $\mathbf{E}_{\mathrm{tot}}(\mathbf{r}_d)$ is the total field at the dipole position (including reflected fields), and ω is the angular frequency.

Denoting by $r^{\uparrow}_{\mathrm{eff}}(k_{\parallel},\omega)$ and $r^{\downarrow}_{\mathrm{eff}}(k_{\parallel},\omega)$ the effective Fresnel reflection coefficients (Fig. S2) of the multilayer stack above and below the tunneling hBN, respectively, the dissipated power of the vertical dipole is (*45*)

$$\mathcal{P}_{\perp} = \frac{\omega}{2}\,\mathrm{Re}\int_{0}^{\infty}\frac{k_{\parallel}}{k_{z,1}}\left(\frac{k_{\parallel}^{2}}{k_{1}^{2}}\,|\mathbf{p}|^{2}\right)\frac{\left(1+r^{\downarrow}_{\mathrm{eff}}e^{ik_{z,1}L}\right)\left(1+r^{\uparrow}_{\mathrm{eff}}e^{-ik_{z,1}L}\right)}{1-r^{\downarrow}_{\mathrm{eff}}r^{\uparrow}_{\mathrm{eff}}e^{2ik_{z,1}L}}\,\mathrm{d}k_{\parallel}. \tag{S2}$$

Here:

- $k_{\|}$: in-plane wavevector,
- $k_1 = k_0\sqrt{\varepsilon_{hBN}}$ with $k_0 = \omega/c$ and ε_{hBN} the dielectric constant of tunneling hBN,
- $k_{z,1} = \sqrt{k_1^2 - k_{\|}^2}$: out-of-plane wavevector in the tunneling hBN,
- L: thickness of the tunneling hBN,
- $r_{\text{eff}}^{\uparrow}, r_{\text{eff}}^{\downarrow}$: effective p-polarized reflection coefficients of the upper and lower sub-stacks.

The normalized LDOS is

$$\frac{\rho}{\rho_0} = \frac{\mathcal{P}_\perp}{\mathcal{P}_0}, \qquad \mathcal{P}_0 = \frac{\omega^4|\mathbf{p}|^2}{12\pi\varepsilon_0 c^3}. \tag{S3}$$

The effective coefficients $r_{\text{eff}}^{\uparrow}$ and $r_{\text{eff}}^{\downarrow}$ are not simple single-interface reflections, but the cumulative reflection response of all the layers above and below the tunneling hBN. They are obtained by applying the standard iterative Fresnel formalism (*44*), which accounts for multiple reflections and interference within each sub-stack. The integrand in the expression for $\mathcal{P}_\perp$ represents the *angular spectrum* of the dissipated power, i.e. the contribution of each in-plane momentum $k_{\|}$ to the total LDOS. Plotting the spectral density

$$\frac{1}{\mathcal{P}_0}\frac{\mathrm{d}\mathcal{P}_\perp}{\mathrm{d}k_{\|}} \tag{S4}$$

for different photon energies and wavevectors provides the k-resolved LDOS maps shown in the main text.

Simulation of the reflection for different thicknesses

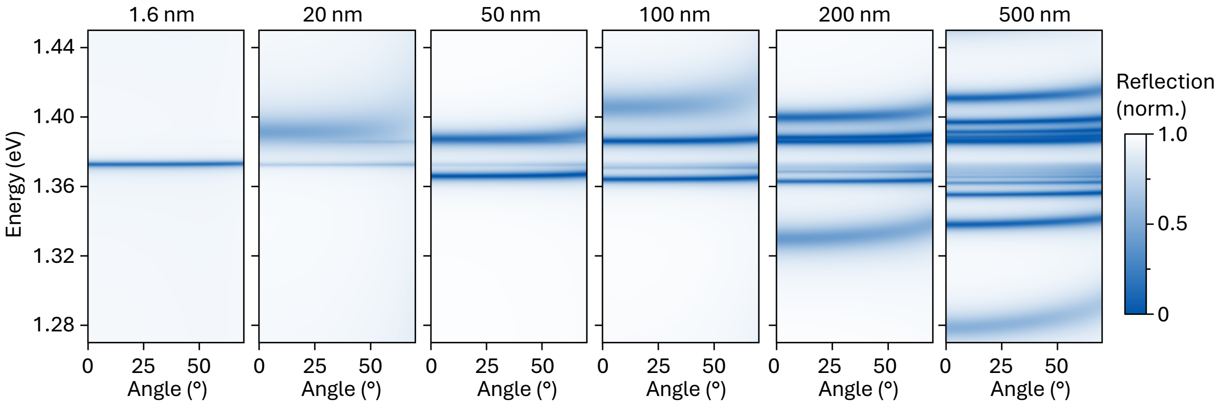

Figure S3: **Thickness dependent reflection.** Simulation of the reflection from CrSBr flakes with different CrSBr thicknesses, and otherwise the same parameter.

Angle-resolved reflectance spectra shown in fig S3 were calculated using a standard transfer-matrix method implemented in MATLAB (*46*). In the simulation, a layered structure hBN (50 nm) - CrSBr - hBN (2 nm)(1000 nm) is interfaced with gold (1000 nm) from one side and air (1000 nm) from the other side. The refractive indices were obtained from (*19*). The simulation for the 1.6 nm thin CrSBr (bilayer) highlights the single resonance of the CrSBr dielectric function used, which evolves to a complex multi-resonance reflection due to the interaction with the optical environment of the stack.

Electroluminescence from graphene-graphene device

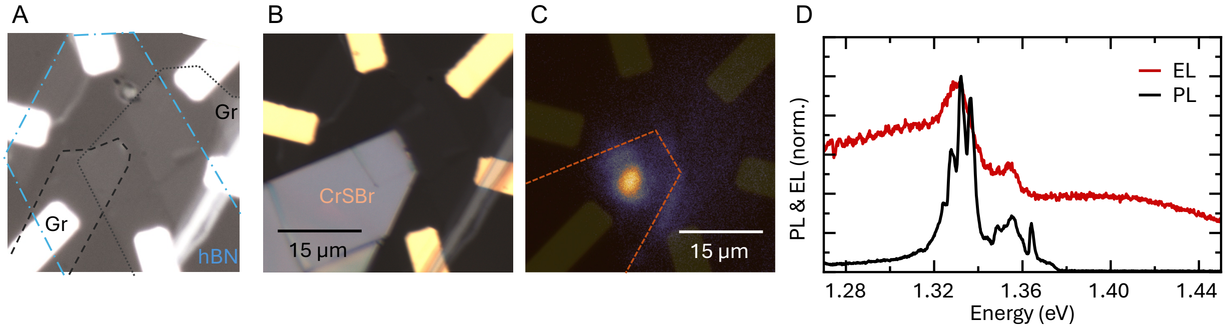

Figure S4: **Emission of graphene-graphene tunnel junction with CrSBr. A** Microscope image of the graphene-hbn-graphene tunnel junction before adding the thick CrSBr, with outlines of the flakes on top. **B** Microscope image of the graphene-graphene tunneling junction with a 250 nm thick CrSBr layer on top. Gold electrodes are contacts for the graphene layers and are not in the active device area. **C** Spatial electroluminescence (EL) from the device, with a microscope image of the electrodes before stacking overlaid. The emission clearly originates only from the overlap area between the two graphene electrodes without any contribution from the gold. Scattered emission from the edges of the flake is additionally visible. **D** Comparison of the EL and PL of the device, recorded from the same spot, reproducing figure 3C of the main manuscript.

Results from a field-effect device

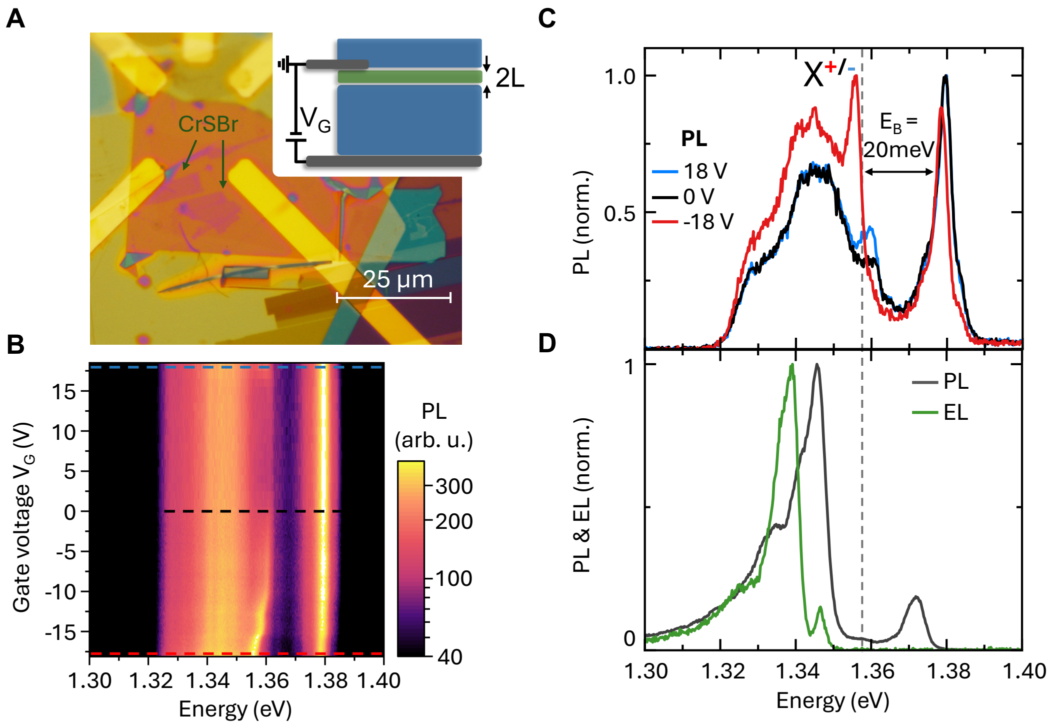

Figure S5: **Charged excitons. A** Microscope image of the field effect device and schematic, with a bilayer CrSBr separated from a graphene gate by a thick (≥ 20 nm) hBN layer. **B** Photoluminescence as function of applied gate voltage, with charged exciton features arising at high voltages. **C** Normalized PL spectra at certain gate voltages, indicated by the coloured lines in B. **D** PL and EL spectra from the bilayer device shown in the main manuscript. The tunneling device from the main manuscript shows slightly different emission characteristics, which we attribute to the direct contact with graphene.

Spatially resolved EL and PL

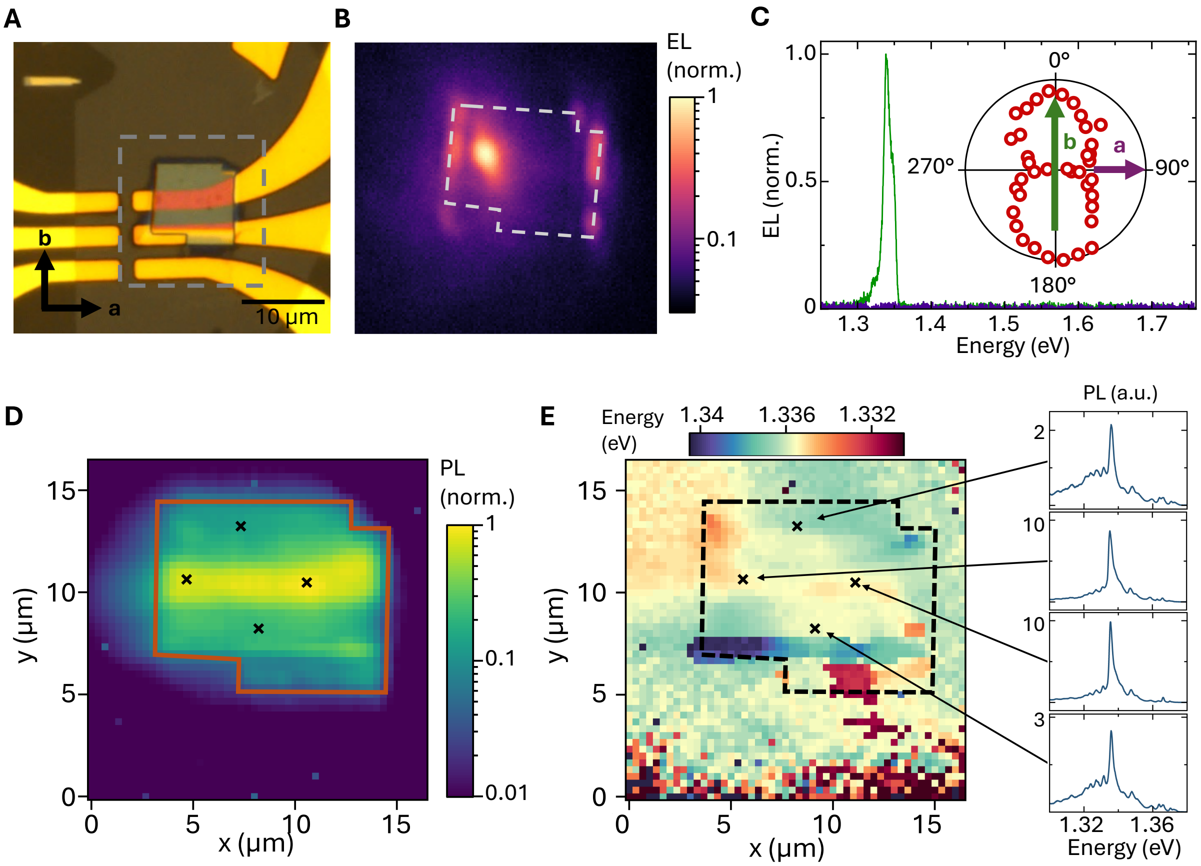

Figure S6: **Characterization of the 104 nm device. A** Optical microscope image of the device. **B** Electroluminescence image under bias from the top electrode. **C** Linear polarization of the EL. **D** Photoluminescence intensity map of the device, the area is outlined in A. **E** Hyperspectral map of the same area, with the energy of the maximum plotted. Selected spectra at relevant positions are marked in D and E.

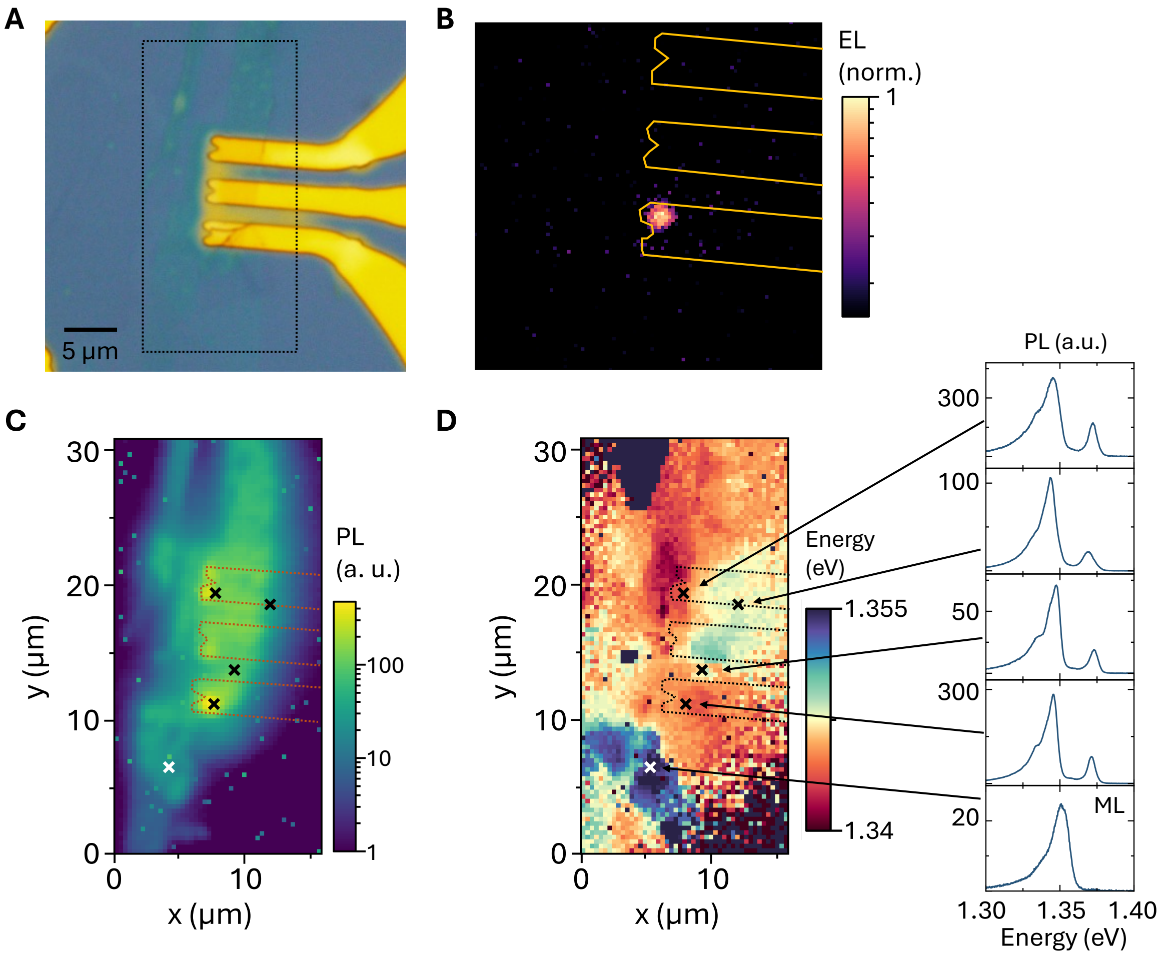

Figure S7: **Characterization of the bilayer device. A** Optical microscope image of the device. **B** Electroluminescence image under bias from the bottom electrode, the electrodes are outlined in yellow. **C** Photoluminescence intensity map of the device, the area is outlined in A. **D** Hyperspectral map of the same area, showing the energy of the PL maximum. Selected spectra at relevant positions are marked in C and D, with the bottom spectra showing the monolayer region (ML).

Additional data from second device

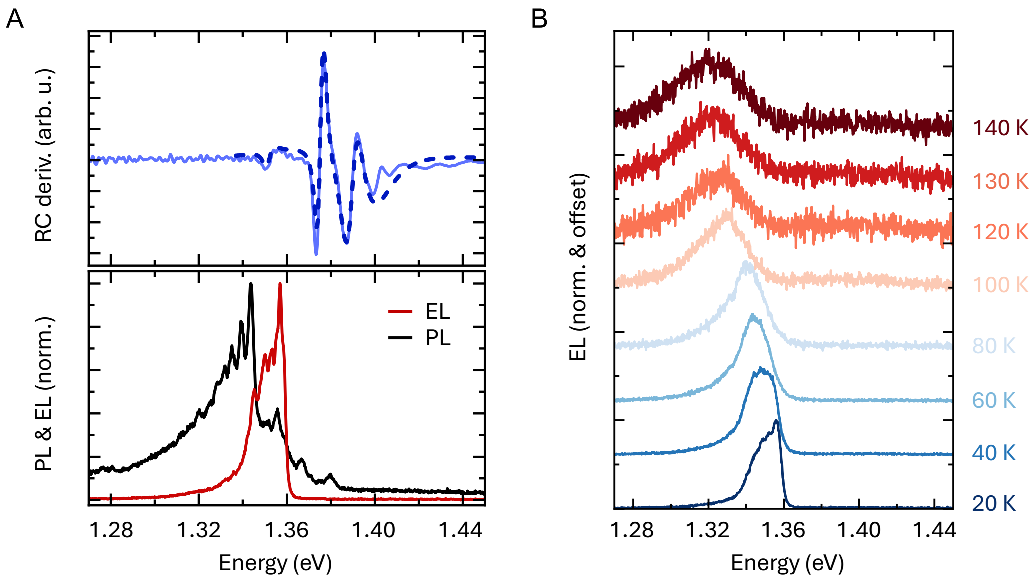

Figure S8: **Comparison of 25 nm thin device.** **(a)** Comparison of reflectance contrast derivative, EL and PL for the 25 nm thin device. The PL and RC derivative already show clear signs of self-hybridization with the cavity mode, while the EL mainly originates from the CrSBr exciton. **b)** Temperature dependence for the same device, additionally showing the stark reduction in EL emission. At 145 K, the device suffered electrical breakdown.

REFERENCES AND NOTES

1. C. Gong, L. Li, Z. Li, H. Ji, A. Stern, Y. Xia, T. Cao, W. Bao, C. Wang, Y. Wang, Z. Q. Qiu, R. J. Cava, S. G. Louie, J. Xia, X. Zhang, Discovery of intrinsic ferromagnetism in two-dimensional van der Waals crystals. *Nature* **546**, 265–269 (2017).

2. B. Huang, G. Clark, E. Navarro-Moratalla, D. R. Klein, R. Cheng, K. L. Seyler, D. Zhong, E. Schmidgall, M. A. McGuire, D. H. Cobden, W. Yao, D. Xiao, P. Jarillo-Herrero, X. Xu, Layer-dependent ferromagnetism in a van der Waals crystal down to the monolayer limit. *Nature* **546**, 270–273 (2017).

3. D. R. Klein, D. MacNeill, J. L. Lado, D. Soriano, E. Navarro-Moratalla, K. Watanabe, T. Taniguchi, S. Manni, P. Canfield, J. Fernández-Rossier, P. Jarillo-Herrero, Probing magnetism in 2D van der Waals crystalline insulators via electron tunneling. *Science* **360**, 1218–1222 (2018).

4. S. Kang, K. Kim, B. H. Kim, J. Kim, K. I. Sim, J. U. Lee, S. Lee, K. Park, S. Yun, T. Kim, A. Nag, A. Walters, M. Garcia-Fernandez, J. Li, L. Chapon, K. J. Zhou, Y. W. Son, J. H. Kim, H. Cheong, J. G. Park, Coherent many-body exciton in van der Waals antiferromagnet $NiPS_3$. *Nature* **583**, 785–789 (2020).

5. K. Hwangbo, Q. Zhang, Q. Jiang, Y. Wang, J. Fonseca, C. Wang, G. M. Diederich, D. R. Gamelin, D. Xiao, J. H. Chu, W. Yao, X. Xu, Highly anisotropic excitons and multiple phonon bound states in a van der Waals antiferromagnetic insulator. *Nat. Nanotechnol.* **16**, 655–660 (2021).

6. F. Dirnberger, R. Bushati, B. Datta, A. Kumar, A. H. MacDonald, E. Baldini, V. M. Menon, Spin-correlated exciton-polaritons in a van der Waals magnet. *Nat. Nanotechnol.* **17**, 1060–1064 (2022).

7. M. Wu, Z. Li, T. Cao, S. G. Louie, Physical origin of giant excitonic and magneto-optical responses in two-dimensional ferromagnetic insulators. *Nat. Commun.* **10**, 2371 (2019).

8. T. Song, X. Cai, M. W. Y. Tu, X. Zhang, B. Huang, N. P. Wilson, K. L. Seyler, L. Zhu, T. Taniguchi, K. Watanabe, M. A. McGuire, D. H. Cobden, D. Xiao, W. Yao, X. Xu, Giant

tunneling magnetoresistance in spin-filter van der Waals heterostructures. *Science* **360**, 1214–1218 (2018).

9. C. Boix-Constant, S. Jenkins, R. Rama-Eiroa, E. J. G. Santos, S. Mañas-Valero, E. Coronado, Multistep magnetization switching in orthogonally twisted ferromagnetic monolayers. *Nat. Mater.* **23**, 212–218 (2024).

10. J. Klein, B. Pingault, M. Florian, M. C. Heißenbüttel, A. Steinhoff, Z. Song, K. Torres, F. Dirnberger, J. B. Curtis, M. Weile, A. Penn, T. Deilmann, R. Dana, R. Bushati, J. Quan, J. Luxa, Z. Sofer, A. Alù, V. M. Menon, U. Wurstbauer, M. Rohlfing, P. Narang, M. Lončar, F. M. Ross, The bulk van der Waals layered magnet CrSBr is a quasi-1D material. *ACS Nano* **17**, 5316–5328 (2023).

11. M. E. Ziebel, M. L. Feuer, J. Cox, X. Zhu, C. R. Dean, X. Roy, CrSBr: An air-stable, two-dimensional magnetic semiconductor. *Nano Lett.* **24**, 4319–4329 (2024).

12. C. Meineke, J. Schlosser, M. Zizlsperger, M. Liebich, N. Nilforoushan, K. Mosina, S. Terres, A. Chernikov, Z. Sofer, M. A. Huber, M. Florian, M. Kira, F. Dirnberger, R. Huber, Ultrafast exciton dynamics in the atomically thin van der Waals magnet CrSBr. *Nano Lett.* **24**, 4101–4107 (2024).

13. K. Lin, X. Sun, F. Dirnberger, Y. Li, J. Qu, P. Wen, Z. Sofer, A. Söll, S. Winnerl, M. Helm, S. Zhou, Y. Dan, S. Prucnal, Strong exciton-phonon coupling as a fingerprint of magnetic ordering in van der Waals layered CrSBr. *ACS Nano* **18**, 2898–2905 (2024).

14. M. Liebich, M. Florian, N. Nilforoushan, F. Mooshammer, A. D. Koulouklidis, L. Wittmann, K. Mosina, Z. Sofer, F. Dirnberger, M. Kira, R. Huber, Controlling Coulomb correlations and fine structure of quasi-one-dimensional excitons by magnetic order. *Nat. Mater.* **24**, 384–390 (2025).

15. N. P. Wilson, K. Lee, J. Cenker, K. Xie, A. H. Dismukes, E. J. Telford, J. Fonseca, S. Sivakumar, C. Dean, T. Cao, X. Roy, X. Xu, X. Zhu, Interlayer electronic coupling on demand in a 2D magnetic semiconductor. *Nat. Mater.* **20**, 1657–1662 (2021).

16. F. L. Ruta, S. Zhang, Y. Shao, S. L. Moore, S. Acharya, Z. Sun, S. Qiu, J. Geurs, B. S. Y. Kim, M. Fu, D. G. Chica, D. Pashov, X. Xu, D. Xiao, M. Delor, X. Y. Zhu, A. J. Millis, X. Roy, J. C. Hone, C. R. Dean, M. I. Katsnelson, M. van Schilfgaarde, D. N. Basov, Hyperbolic exciton polaritons in a van der Waals magnet. *Nat. Commun.* **14**, 8261 (2023).

17. T. Jungwirth, J. Sinova, A. Manchon, X. Marti, J. Wunderlich, C. Felser, The multiple directions of antiferromagnetic spintronics. *Nat. Phys.* **14**, 200–203 (2018).

18. Y. Chen, K. Samanta, N. A. Shahed, H. Zhang, C. Fang, A. Ernst, E. Y. Tsymbal, S. S. P. Parkin, Twist-assisted all-antiferromagnetic tunnel junction in the atomic limit. *Nature* **632**, 1045–1051 (2024).

19. F. Dirnberger, J. Quan, R. Bushati, G. M. Diederich, M. Florian, J. Klein, K. Mosina, Z. Sofer, X. Xu, A. Kamra, F. J. García-Vidal, A. Alù, V. M. Menon, Magneto-optics in a van der Waals magnet tuned by self-hybridized polaritons. *Nature* **620**, 533–537 (2023).

20. D. Bajoni, E. Semenova, A. Lemaître, S. Bouchoule, E. Wertz, P. Senellart, J. Bloch, Polariton light-emitting diode in a GaAs-based microcavity. *Phys. Rev. B* **77**, 113303 (2008).

21. A. Canales, D. G. Baranov, T. J. Antosiewicz, T. Shegai, Abundance of cavity-free polaritonic states in resonant materials and nanostructures. *J. Chem. Phys.* **154**, 024701 (2021).

22. S. Papadopoulos, L. Wang, T. Taniguchi, K. Watanabe, L. Novotny, Energy transfer from tunneling electrons to excitons. arXiv:2209.11641 (2022).

23. L. Wang, S. Papadopoulos, F. Iyikanat, J. Zhang, J. Huang, T. Taniguchi, K. Watanabe, M. Calame, M. L. Perrin, F. J. García de Abajo, L. Novotny, Exciton-assisted electron tunnelling in van der Waals heterostructures. *Nat. Mater.* **22**, 1094–1099 (2023).

24. M. Parzefall, P. Bharadwaj, L. Novotny, in *Antenna-Coupled Tunnel Junctions* (Springer International Publishing, 2017), pp. 211–236.

25. M. Parzefall, P. Bharadwaj, A. Jain, T. Taniguchi, K. Watanabe, L. Novotny, Antenna-coupled photon emission from hexagonal boron nitride tunnel junctions. *Nat. Nanotechnol.* **10**, 1058–1063 (2015).

26. J. Kern, R. Kullock, J. Prangsma, M. Emmerling, M. Kamp, B. Hecht, Electrically driven optical antennas. *Nat. Photon.* **9**, 582–586 (2015).

27. H. Qian, S. W. Hsu, K. Gurunatha, C. T. Riley, J. Zhao, D. Lu, A. R. Tao, Z. Liu, Efficient light generation from enhanced inelastic electron tunnelling. *Nat. Photon.* **12**, 485–488 (2018).

28. T. Förster, Zwischenmolekulare energiewanderung und fluoreszenz. *Ann. Phys.* **437**, 55–75 (1948).

29. P. J. Zomer, M. H. D. Guimarães, J. C. Brant, N. Tombros, B. J. van Wees, Fast pick up technique for high quality heterostructures of bilayer graphene and hexagonal boron nitride. *Appl. Phys. Lett.* **105**, 013101 (2014).

30. E. J. Telford, A. H. Dismukes, R. L. Dudley, R. A. Wiscons, K. Lee, D. G. Chica, M. E. Ziebel, M. G. Han, J. Yu, S. Shabani, A. Scheie, K. Watanabe, T. Taniguchi, D. Xiao, Y. Zhu, A. N. Pasupathy, C. Nuckolls, X. Zhu, C. R. Dean, X. Roy, Coupling between magnetic order and charge transport in a two-dimensional magnetic semiconductor. *Nat. Mater.* **21**, 754–760 (2022).

31. K. Torres, A. Kuc, L. Maschio, T. Pham, K. Reidy, L. Dekanovsky, Z. Sofer, F. M. Ross, J. Klein, Probing defects and spin-phonon coupling in CrSBr via resonant raman scattering. *Adv. Funct. Mater.* **33**, 2211366 (2023).

32. Y. Shao, F. Dirnberger, S. Qiu, S. Acharya, S. Terres, E. J. Telford, D. Pashov, B. S. Y. Kim, F. L. Ruta, D. G. Chica, A. H. Dismukes, M. E. Ziebel, Y. Wang, J. Choe, Y. J. Bae, A. J. Millis, M. I. Katsnelson, K. Mosina, Z. Sofer, R. Huber, X. Zhu, X. Roy, M. van Schilfgaarde, A. Chernikov, D. N. Basov, Magnetically confined surface and bulk excitons in a layered antiferromagnet. *Nat. Mater.* **24**, 391–398 (2025).

33. F. Tabataba-Vakili, H. P. G. Nguyen, A. Rupp, K. Mosina, A. Papavasileiou, K. Watanabe, T. Taniguchi, P. Maletinsky, M. M. Glazov, Z. Sofer, A. S. Baimuratov, A. Högele, Doping-control of excitons and magnetism in few-layer CrSBr. *Nat. Commun.* **15**, 4735 (2024).

34. A. Kuzmina, M. Parzefall, P. Back, T. Taniguchi, K. Watanabe, A. Jain, L. Novotny, Resonant light emission from graphene/hexagonal boron nitride/graphene tunnel junctions. *Nano Lett.* **21**, 8332–8339 (2021).

35. S. Shan, J. Huang, S. Papadopoulos, R. Khelifa, T. Taniguchi, K. Watanabe, L. Wang, L. Novotny, Overbias photon emission from light-emitting devices based on monolayer transition metal dichalcogenides. *Nano Lett.* **23**, 10908–10913 (2023).

36. G. Froehlicher, E. Lorchat, S. Berciaud, Charge versus energy transfer in atomically thin graphene-transition metal dichalcogenide van der Waals heterostructures. *Phys. Rev. X* **8**, 011007 (2018).

37. S. Dong, S. Beaulieu, M. Selig, P. Rosenzweig, D. Christiansen, T. Pincelli, M. Dendzik, J. D. Ziegler, J. Maklar, R. P. Xian, A. Neef, A. Mohammed, A. Schulz, M. Stadler, M. Jetter, P. Michler, T. Taniguchi, K. Watanabe, H. Takagi, U. Starke, A. Chernikov, M. Wolf, H. Nakamura, A. Knorr, L. Rettig, R. Ernstorfer, Observation of ultrafast interfacial Meitner-Auger energy transfer in a Van der Waals heterostructure. *Nat. Commun.* **14**, 5057 (2023).

38. D. J. Rizzo, E. Seewald, F. Zhao, J. Cox, K. Xie, R. A. Vitalone, F. L. Ruta, D. G. Chica, Y. Shao, S. Shabani, E. J. Telford, M. C. Strasbourg, T. P. Darlington, S. Xu, S. Qiu, A. Devarakonda, T. Taniguchi, K. Watanabe, X. Zhu, P. J. Schuck, C. R. Dean, X. Roy, A. J. Millis, T. Cao, A. Rubio, A. N. Pasupathy, D. N. Basov, Engineering anisotropic electrodynamics at the graphene/CrSBr interface. *Nat. Commun.* **16**, 1853 (2025).

39. B. Yang, B. Bhujel, D. G. Chica, E. J. Telford, X. Roy, F. Ibrahim, M. Chshiev, M. Cosset-Chéneau, B. J. Wees, Electrostatically controlled spin polarization in graphene-CrSBr magnetic proximity heterostructures. *Nat. Commun.* **15**, 4459 (2024).

40. A. Beer, K. Zollner, C. Serati de Brito, P. E. Faria Junior, P. Parzefall, T. S. Ghiasi, J. Ingla-Aynés, S. Mañas-Valero, C. Boix-Constant, K. Watanabe, T. Taniguchi, J. Fabian, H. S. J. van der Zant, Y. Galvão Gobato, C. Schüller, Proximity-induced exchange interaction and prolonged valley lifetime in $MoSe_2$/CrSBr van-der-Waals heterostructure with orthogonal spin textures. *ACS Nano* **18**, 31044–31054 (2024).

41. Y. Huo, S. Li, L. Yan, N. Li, J. Zou, J. He, T. Zhou, T. Frauenheim, S. Tretiak, L. Zhou, Ultrafast laser-induced spin dynamics in all-semiconductor ferromagnetic CrSBr-phosphorene heterostructures. *J. Phys. Chem. Lett.* **16**, 782–788 (2025).

42. Y. Wang, S. Yang, L. Huang, Y. Ran, P. Gu, X. Huang, K. Watanabe, T. Taniguchi, Z. Chen, Y. Ye, Polarized electroluminescence with magnetic spectral tuning in van der Waals magnet CrSBr. arXiv:2506.06734 (2025).

43. F. Qin, H. Liu, A. Yang, Y. Liu, X. Wang, Y. Sun, X. Zhou, Z. Sofer, J. Zhou, X. Liu, S. Liu, V. L. Zhang, X. Liu, W. Gao, T. Yu, Spin light-emitting devices in a 2D magnet. arXiv:2508.00572 (2025).

44. L. Novotny, B. Hecht, *Principles of Nano-Optics* (Cambridge Univ. Press, 2012).

45. G. Ford, W. Weber, Electromagnetic interactions of molecules with metal surfaces. *Phys. Rep.* **113**, 195–287 (1984).

46. H. Benisty, R. Stanley, M. Mayer, Method of source terms for dipole emission modification in modes of arbitrary planar structures. *J. Opt. Soc. Am. A* **15**, 1192–1201 (1998).
